# The Ingenious Synthesis of a Nitro-Free Insensitive High-Energy Material Featuring Face-to-Face and Edge-to-Face π-Interactions

**DOI:** 10.3389/fchem.2019.00559

**Published:** 2019-08-07

**Authors:** Lianjie Zhai, Fuqiang Bi, Huan Huo, Yifen Luo, Xiangzhi Li, Sanping Chen, Bozhou Wang

**Affiliations:** ^1^State Key Laboratory of Fluorine & Nitrogen Chemicals, Xi'an Modern Chemistry Research Institute, Xi'an, China; ^2^College of Chemistry and Materials Science, Northwest University, Xi'an, China

**Keywords:** energetic materials, detonation performances, *N*-heterocycles, π-interactions, crystal structure

## Abstract

Density, detonation property, and sensitivity may be the most valued features when evaluating an energetic material. By reasoning structure–property relationships, a nitro-free planar energetic material with high nitrogen and oxygen content, 7-hydroxy-difurazano[3,4-*b*:3′,4′-*f*]furoxano[3″,4″-*d*]azepine (**4**), was synthesized using a unique and facile approach. The structure was fully characterized by IR and NMR spectra, elemental analysis, differential scanning calorimetry (DSC), and single-crystal X-ray diffraction. The expected properties of **4**, including a high density of 1.92 g cm^−3^, high detonation velocity of 8,875 m s^−1^, and low mechanical sensitivities (impact sensitivity, 21 J and friction sensitivity, >360 N), confirm our strategy. Interestingly, the single-crystal structures of **4** reveal expected face-to-face and edge-to-face π-interactions in the crystal stacking. The remarkable differences in crystal stacking of **4** provide unequivocal evidence that face-to-face π-π interactions contribute significantly to closer assembly and higher density.

## Introduction

At the early stages of energetic materials, chemists mainly focused on designing and synthesizing the polynitro compounds, such as 1,3,5-trinitro-1,3,5-triazinane (RDX), 1,3,5,7-tetranitro-1,3,5,7-tetrazocane (HMX), 2,4,6,8,10,12-hexanitro-2,4,6,8,10, 12-hexaazaisowurtzitane (CL-20), and octanitrocubane (ONC) (Badgujar et al., 2008; Klapötke, 2012). Nitro group has been playing a critical role in the developing of energetic materials since nitroglycerin (NG) and 2,4,6-trinitrotoluene (TNT) were widely used for the purpose of military and civilization. The contributions of nitro group primarily consist of enhancing the oxygen balance and density, leading to an increase in the detonation performances. Although the detonation performances of nitro compounds increase greatly as the increasing number of nitro groups, the synthesis is becoming more difficulty due to multiple steps. More than that, compounds with an excess of nitro groups always exhibit high sensitivities and low thermal decomposition temperatures arising from their congested molecule structures (Vishnevskiy et al., 2017). Along with growing concerns about the safety issues, more considerable effort has been devoted to pursuing the highly insensitive high-energy materials (Klapötke and Witkowski, 2016; Tian et al., 2017; Wang Y. et al., 2018). In most cases, effects of the amounts of nitro groups on sensitivity, density and oxygen balance always are contradictory, which makes the design and synthesis of new superior HEDMs an interesting and formidable challenge (Gao and Shreeve, 2011; Li et al., 2013; Wang P. C. et al., 2018).

From a molecular design perspective, detonation performances including detonation velocity and pressure are highly dependent on the density, heat of formation and oxygen balance, which are directly determined by the composition and structure of the compound. Therefore, energetic compounds featuring high density, positive heat of formation, and good oxygen balance but containing no nitro group are also likely to exhibit both superior denotation performances and low sensitivities. Thus, searching for nitro-free energetic compounds with these features may become a promising strategy to generate new insensitive high-energy materials.

**Figure 1:**
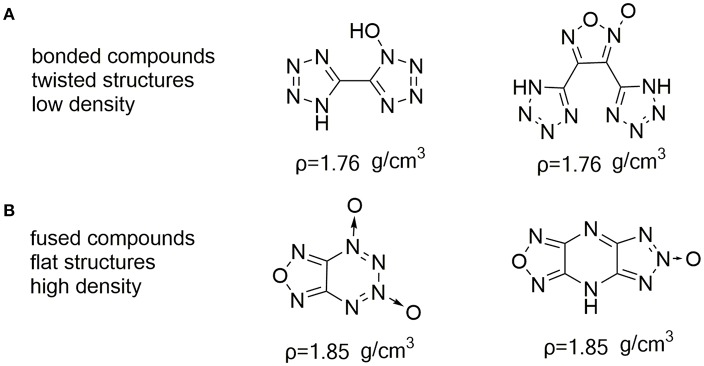
Nitro-free C-C bonded **(A)**, and fused **(B)** compounds consisting of nitrogen- and oxygen-containing heterocycles.

Nitrogen- and oxygen-containing heterocycles based on furazan (Sheremetev et al., 1998; Huang et al., 2012; Zhai et al., 2015b), furoxan (Fischer et al., 2014a; He et al., 2018; Liu et al., 2018; Khakimov et al., 2019), triazole *N*-oxides (Dippold and Klapötke, 2013; Zhang et al., 2013) and tetrazole *N*-oxide (Fischer et al., 2012; He et al., 2016) are promising compounds that fulfill above-mentioned requirements. Not only does the high nitrogen increases the heat of formation due to inherently C–N and N–N bonds, the oxygen of N–O bonds contained in the heterocycles also serves the same function as nitro groups do. Especially, the *N*-hydroxy has been shown to usually result in an increased density, and oxygen balance, but also lower its sensitivity due to forming hydrogen bonds. However, the majority of nitro-free heterocyclic compounds, such as C-C bonded molecules usually result in a lower density (Figure 1A) (Fischer et al., 2014b; Zhai et al., 2015a). Recently it has been observed by Shreeve group that energetic materials with parallel face-to-face crystal stacking are found to show higher densities and relative low sensitivities because of π-stacked interaction and free interlayer sliding (Yin et al., 2016; Liu et al., 2019). This type is strikingly represented by fused energetic compounds (Figure 1B) (Thottempudi et al., 2014; Tang et al., 2017; Hu et al., 2018; Wang et al., 2019).

Based on the analysis above, we are aimed at designing and synthesizing high-performance insensitive materials that have the following structure characteristics: (1) a near-perfect planar molecular being able to form face-to-face stacking to achieve a high density; (2) high nitrogen and oxygen content leading to high heat of formation and good oxygen balance; (3) no nitro within it to achieve a low sensitivity. With these in mind, we were interested in some furazan/furoxan-based planar compounds containing *N*- hydroxy group. Recently, new cyclic planar azepine/oxepine compounds containing furazan/furoxan fragment, such as difurazano[3,4-*b*:3′,4′-*f*]furoxano[3″,4″-*d*]oxepine, 7*H*-difurazano[3,4-*b*:3′,4′-*f*]furoxano[3″,4″'-*d*]azepine, etc., were synthesized by Russian researchers and our group (Stepanov et al., 2012; Zhou et al., 2012). The fascinating structures and promising properties attracted our attention to the 7-hydroxy-difurazano[3,4-*b*:3′,4′-*f*]furoxano[3″,4″-*d*]azepine (**4**), which is expected to higher density and oxygen balance due to introduction of *N*- hydroxy. Herein, we now report the results on the ingenious synthesis, full characterization, energetic properties of **4**.

## Materials and Methods

*Caution:* While we have experienced no difficulties in syntheses and characterization of these materials, proper protective measures should be used. Manipulations must be carried out in a hood behind a safety shield. Eye protection and leather gloves must be worn.

### General Methods

The reagents were available commercially and were used as purchased without further purification. ^1^H, ^13^C, and ^15^N NMR spectra were recorded on 500 MHz (Bruker AVANCE 500) nuclear magnetic resonance spectrometers by using [D_6_]DMSO as solvent. The decomposition points (onset) were obtained on a differential scanning calorimeter (TA Instruments Company, Model DSC-Q200) at a flow rate of 50 mL min^−1^. About 0.3 mg of the sample was sealed in aluminum pans for DSC at a rate of 5°C min^−1^. Infrared spectra were obtained from KBr pellets on a Nicolet NEXUS870 Infrared spectrometer in the range of 4,000–400 cm^−1^. Elemental analyses (C, H, and N) were performed on a VARI-El-3 elementary analysis instrument. The impact and friction sensitivities were determined following the BAM method.

### Computational Details

All quantum chemical calculations were carried out using the Gaussian 09 (Revision A.02) program package (Frisch et al., 2009) and visualized by GaussView 5.08 (Dennington and Millam, 2009). The enthalpies (*H*°) and free energies (*G*°) were calculated using the complete basis set method (CBS-4M) based on X-ray diffraction data, in order to obtain accurate (Ochterski et al., 1996; Montgomery et al., 2000). The enthalpies of the gas-phase species were estimated according to the atomization energy method (Curtiss et al., 1997). The solid state enthalpy of formation can be estimated by subtracting the heats of sublimation from gas phase heats of formation. The heat of sublimation can be estimated with Trouton's rule (Westwell et al., 1995) according to Equation 1, where *T* represents either the melting point or the decomposition temperature when no melting occurs prior to decomposition:

(1) $$\begin{array}{r} \Delta H_{\text{sub}}=188/\text{Jmo}{\text{l}}^{-1}{\text{K}}^{-1}\times T \end{array}$$

### Crystallographic Measurements

Single-crystal X-ray diffraction data were collected with on a Bruker SMART Apex II CCD X-ray diffractometer equipped with a graphite-monochromatized Mo*K*α radiation (λ = 0.71073 Å). The structures were solved either with SHELXS-97 (Sheldrick, 1997a), refined with SHELXL-97 (Sheldrick, 1997b). The full-matrix least-squares refinement on *F*^2^ included atomic coordinates and anisotropic thermal parameters for all non-H atoms. The H atoms were found and refined. CCDC-1911348 (for **4·**MeOH), and−1911347 (for **4·**H_2_O) contain the supplementary crystallographic data for this paper. These data can be obtained free of charge from The Cambridge Crystallographic Data Center via www.ccdc.cam.ac.uk/data_request/cif.

### Synthesis

Compounds **2** was prepared were prepared with a high yield according to the literature procedure (Tsyshevsky et al., 2015).

7*H*-difurazano[3,4-*b*:3′,4′-*f*]furoxano[3″,4″-*d*]azepine (**3**): 3,4-Bis(3-nitrofurazan-4-yl)furoxan (3.1 g, 10 mmol) is dissolved in 50 mL of CH_3_CN at 10°C, to which 0.65 g (10 mmol) of 50% hydroxylamine solution was added dropwise within 30 min. After stirred for 2 h, the solvent was removed under reduced pressure, and the resident was recrystallized from ethanol/H_2_O (v/v = 1:1), 1.59 g (67.6%) colorless crystals of **3** were obtained. DSC (5°C min^−1^): 227.8°C (m.p.), 273.9°C (onset); IR (KBr): ṽ = 3293, 3215, 3113, 3080, 2976, 2817, 2705, 1655, 1633, 1615, 1572, 1553, 1492, 1462, 1443, 1393, 1359, 1225, 1152, 1062, 996, 970, 897, 879, 820, 781, 717 cm^−1^; ^1^H NMR ([D_6_]DMSO, 500 MHz, 25°C, TMS): δ = 12.56 (s, H, NH) ppm; ^13^C NMR ([D_6_]DMSO, 125 MHz, 25°C, TMS): δ = 152.44, 151.99, 145.60, 138.10, 135.83, 106.40 ppm; elemental analysis calcd (%) for C_6_HN_7_O_4_: C 30.65, H 0.43, N 41.70; found, C 30.77, H 0.39, N 40.92.

7-Hydroxy-difurazano[3,4-*b*:3′,4′-*f*]furoxano[3″,4″-*d*] azepine (**4**): 3,4-Bis(3-nitrofurazan-4-yl)furoxan (3.1 g, 10 mmol) is dissolved in 15 mL of CH_3_CN, to which 0.65 g (10 mmol) of 50% hydroxylamine solution was added dropwise within 30 min at −10°C. After 1 h the precipitate was filtered and air-dried to yield **4** (1.12 g, 44.7%) as a light yellow solid. DSC (5°C min^−1^): 160.6°C (onset); IR (KBr): ṽ = 3282, 2087, 2454, 1658, 1637, 1608, 1588, 1555, 1528, 1482, 1440, 1395, 1355,1173, 1054, 996, 969, 939, 851, 797, 736 cm^−1^; ^1^H NMR ([D_6_]DMSO, 500 MHz, 25°C, TMS): δ = 7.81 (s, OH) ppm; ^13^C NMR ([D_6_]DMSO, 125 MHz, 25°C, TMS): δ = 155.08, 154.68, 144.98, 135.72, 133.66, 105.96 ppm; ^15^N NMR ([D_6_]DMSO, 50.6 MHz, 25°C, MeNO_2_): δ = −25.2, −8.7, −5.6, −3.1, 32.0, −32.7 −253.3 ppm; elemental analysis calcd (%) for C_6_HN_7_O_5_: C 28.70, H 0.40, N 39.04; found, C 28.31, H 0.45, N 38.64.

## Results and Discussion

### Synthesis

**Figure 2:**
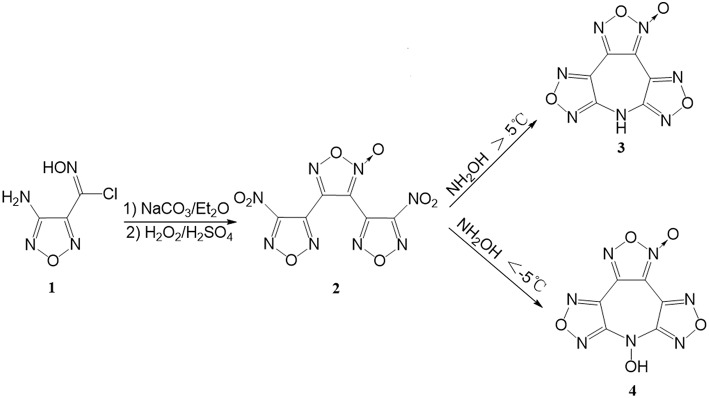
The interesting reaction toward **4**.

The synthetic pathway to **4** is shown in Figure 2. 3,4-Bis(3-nitrofurazan-4-yl)furoxan (**2**) was readily prepared according to a known procedure (Tsyshevsky et al., 2015). We initially intended to prepare **4** by treatment of acetonitrile solution of **2** with 50% aqueous NH_2_OH undergoing an amino-substitution annulation at the temperature higher than 5°C. However, what amazed us was that the product separated from the reaction solution ultimately proved to be 7*H*-difurazano[3,4-*b*:3′,4′-*f*]furoxano[3″,4″-*d*]azepine (**3**). Many attempts to synthesize the title compound performed in other various solvents (THF, MeOH, DMF, acetone, and ethyl acetate) also failed. On the flip side, the unexpected product **3** may indicate the success of amino-substitution annulation between **2** and NH_2_OH, which led us to believe that the title compound **4** was likely to have formed as an intermediate. Not unexpectedly, a new compound, i.e., **4**, was detected immediately by thin layer chromatography when NH_2_OH aqueous was added, but it disappears quickly as the reaction went on. The reason might be due to N-OH reactivity of **4**. Therefore, in order to effectively control the cyclization reaction and easily isolate **4** from the reaction mixture, low temperature and minimal amount of CH_3_CN are employed during the reaction. Fortunately, **4** was successfully obtained and easily isolated by filtering, and no **3** was detected in the reaction mixture.

### Spectroscopy

**Figure 3:**
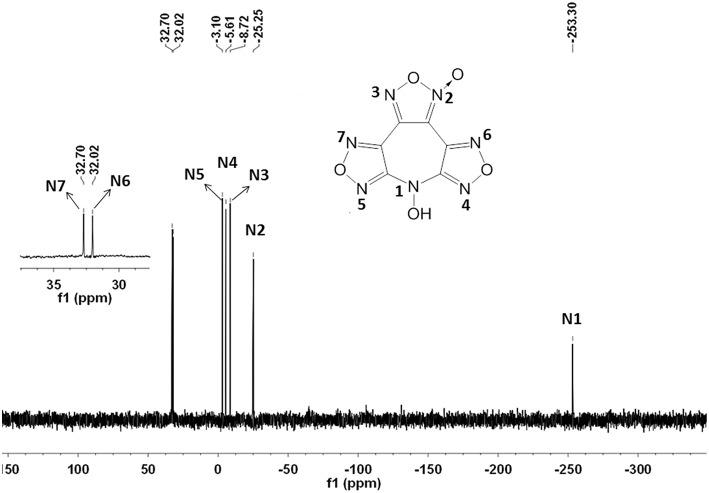
^15^N spectrum of compound **4** in [D6]DMSO.

The structure of **4** was investigated by IR and NMR spectroscopy, elemental analysis, and X-ray diffraction. The ^1^H NMR spectrum exhibits one sharp single peak at 7.81 ppm for the *N*-hydroxy groups (Figure S1). As expected, six different signals for the chemically different carbon atoms were observed in the ^13^C NMR spectrum (Figure S2). Among them, the furoxan exhibits two resonances at 105.96 and 144.98 ppm for the carbon bonded to *N*–oxide and the other carbon, respectively. The residual four resonances at 155.08, 154.68, 135.72, and 133.66 ppm were assigned to the furazan carbons. In addition, the ^15^N NMR spectrum is shown in Figure 3. The signal of N-OH can be assigned to the resonance peak at highest field (δ≈-253.3 ppm). And the nitrogen resonances of furazan and furoxan observed at shifts of −25.2 (N2), −8.7 (N3), −5.6 (N4), −3.1(N5), 32.0 (N6), and 32.7 (N7) ppm were also successfully assigned based on assigned based on the literatures, and confirmed by GIAO NMR calculation (Knijn et al., 2010).

### Single-Crystal X-Ray Structure Analysis

Many attempts to cultivate the single crystals of **4** for XRD were performed, and single crystals **4·**MeOH and **4·**H_2_O were obtained by slow evaporation of methanol solutions and acetone aqueous at room temperature, respectively. Crystallographic data and parameters are given in Table S1.

**Figure 4:**
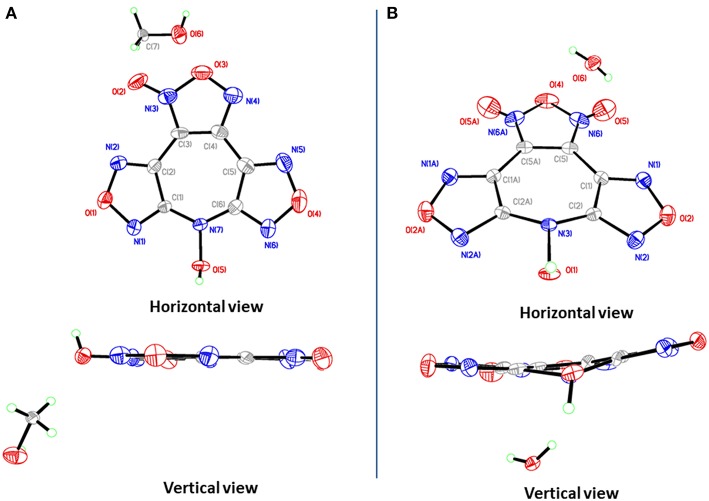
Single-crystal X-ray structures of **4**·MeOH **(A)** and **4**·H_2_O **(B)**.

**Figure 5:**
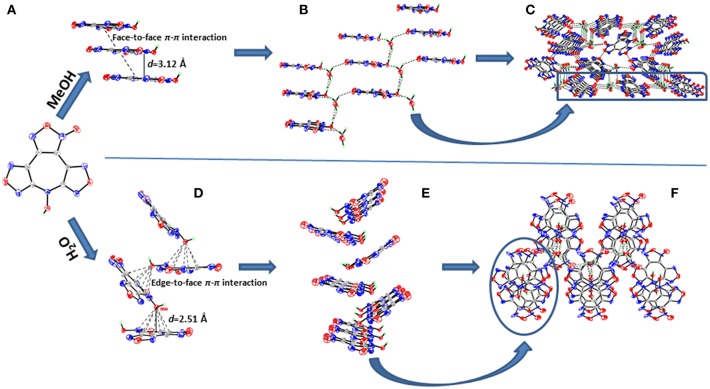
**(A)** Parallel face-to-face arrangements within **4·**MeOH. **(B)** Hydrogen-bonding interactions between MeOH and adjacent **4**. **(C)** Layered three-dimensional packing of **4·**MeOH. **(D)** Edge-to-face π-π interactions within **4·**H_2_O. **(E)** Zigzag-shaped two-dimensional sheet within **4·**H_2_O. **(F)** Three-dimensional packing of **4·**H_2_O.

**4·**MeOH crystallizes in the orthorhombic space group *Pna*2_1_ with four formula units in the unit cell. Surprisingly, though the crystals contain one methanol molecule, it still has a remarkable high calculated density of 1.887 g cm^−3^ at 296 K. The furoxan ring and the furazan rings in **4** are completely in one plane with the dihedral angle of 0°, including the *N*-hydroxy group (Figure 4A). The average distances of C–N and N–O bonds within the furoxan/furazan rings are 1.31 Å and 1.39 Å, respectively, which are all in the range of formal C–N and N–O single and double bonds (C–N: 1.47 Å, 1.22 Å; N–O: 1.46 Å, 1.21 Å) (Allen et al., 1987). The bond lengths of C1–N7 (1.37 Å) and C6–N7 (1.35Å) are slightly longer than the C–N distances of furoxan/furazan rings, but significantly shorter than the distance of the C–N single bond. Additional, the O5–N7 bond length (1.39 Å) of the *N*-hydroxy group is comparable with the O–N distances in the furoxan/furazan ring. These findings support the presence of a delocalized large π-system in the seven-membered azepine ring of **4**. As expected, the face-to-face stacking between the flat molecules provides key driving force to form lamellate one-dimensional molecular chain (Figure 5A). The distances between the molecules of was measured to be 3.12 Å, and distinctly shorter than typical parameters of aromatic face-to-face π-interactions (<4.00 Å). The dihedral angle is exactly 0°, which further confirms parallel face-to-face arrangements. Finally, the hydrogen-bonding interactions (Figure 5B) between MeOH molecules and adjacent **4** molecules further drives the adjacent chains forming a three-dimensional layer assembly (Figure 5C).

**4·**H_2_O crystallizes in the same space group as **4·**MeOH with four formula units in the unit cell and a density of 1.797 g cm^−3^ at 293 K. However, the most striking difference between the crystal structures **4·**MeOH and **4·**H_2_O is observed in their molecular conformation of **4**. The molecule in **4·**H_2_O is distinctly twisted rather than a flat configuration, which are not consistent with our assumption about its packing pattern (Figure 4B). Both the dihedral angles between furoxan ring and the furazan rings are 8.5°, and the dihedral angle between two furazan rings reaches 15.7°. After indepth analysis of the crystal structure, we found that each oxygen atom (O1) of *N*-hydroxy group is intensively involved in an interaction with the π-electrons of the overlying seven-membered azepine ring, which leads to a zigzag-shaped one-dimensional chain (Figures 5D,E). The contact distances between O1 and the carbon atoms in azepine ring are in the range from 2.91 to 3.12 Å. And the distance between O1 and azepine ring plane is 2.51 Å, indicating an intense edge-to-face π-π interactions. All water molecules in **4·**H_2_O perform a bridging connection functions by the formation of numerous hydrogen bonds. Each **4** molecules interacted with three surrounding water molecules through three significant hydrogen bonds [O1^i^-H1A^i^···O6^i^: 2.629(163.2) Å; O6–H6B···O5^ii^: 2.969(139.3) Å; O6–H6A···N1^iii^: 3.026(170.7) Å. Symmetry codes: i: −1/2 + x, 3/2–y, z; ii: 1–x, 2–y, −1/2+z; iii: 1–x, 2–y, ½ + z] to form a 2D wave-like layer structure. However, hydrogen bonds do not participate in forming the 3D structure, and the non-coplanar structure of **4** also indicates no face-to-face π-interactions between the layers. In fact, it is the interaction between oxygen atom (O1) of *N*-hydroxy group and the π-electrons of the azepine ring that further assembles to form to 3D structure (Figure 5F). Hence, stacking differences of **4·**MeOH and **4·**H_2_O provide unequivocal evidence that face-to-face π-π interactions of planar molecule contribute significantly to closer assembly and higher density.

### Physical and Energetic Properties

The physical and energetic properties of **4** are summarized in Table 1. It should be noted that dried **4** do not absorb water from the air. The density of **4** was measured by using a gas pycnometer and found to be 1.92 g cm^−3^, which is expected based on the high density (1.887 g cm^−3^) of **4·**MeOH. Compared with those polynitro compounds, such as TNT, triaminotrinitrobenzene (TATB), RDX, and 1,1-diamino-2,2-dinitroethene (FOX-7), the remarkable high density of **4** confirms the strategy that nitro-free flat heterocycles molecules also could achieve high density. As expected, **4** shows a fairly low impact sensitivity (21 J) and friction sensitivity (>360 N), which are much superior to those of TNT and RDX, comparable to FOX-7, but higher than typical insensitivity explosives TATB. Deriving from one furoxan and two furazan rings, **4** has much higher positive heats of formation (714.6 kJ mol^−1^) than those of TNT, TATB, RDX, and FOX-7. Though **4** does not has nitro group within the molecular structure, it has a good oxygen balance (Ω_(CO)_ = −9.5%) compared with those of TNT and TATB. However, the thermal decomposition temperature of **4** is slightly low with the onset decomposition temperature at 160.6°C, as shown in Figure S3. The detonation properties of **4** were evaluated by using the EXPLO5 6.04 program (Sućeska, 2017). The calculated detonation velocity and detonation pressure of **4** are 8,875 m s^−1^ and 35.0 GPa, respectively, which are much superior to those of TNT (*v*_D_ = 7,017 m s^−1^, *P* = 21.1 GPa) and TATB (*v*_D_ = 7,982 m s^−1^, *P* = 30 GPa). The detonation performances of **4** are comparable to those of RDX (*v*_D_ = 8,823 m s^−1^, *P* = 35.1 GPa), but slightly lower that than that of FOX-7 (*v*_D_ = 9,000 m s^−1^, *P* = 35.9 GPa). Noteworthy, these performances make it as one of promising insensitive material with good detonation performances.

**Table 1:** Physical and energetic properties of **4** compared with polynitro compounds TNT, TATB, RDX, and FOX-7.

	**4**	**TNT**	**TATB**	**RDX**	**FOX-7**
Formula	C_6_HN_7_O_5_	C_7_H_5_N_3_O_6_	C_6_H_6_N_6_O_6_	C_3_H_6_N_6_O_6_	C_2_H_4_N_4_O_4_
*M*/g mol^−1^	251.1	227.1	258.1	222.1	148.1
IS/J^[a]^	21	15	>40	7.4	24.7
FS/N^[b]^	>360	>360	>360	120	>360
N + O/%^[c]^	70.9	60.7	69.7	81.0	81.1
Ω(CO)/%^[d]^	−9.5	−24.6	−18.6	0	0
*T*_dec_/°C^[e]^	160.6	295	360	210	220
*ρ/*g cm^−3^^[f]^	1.92	1.65	1.94	1.80	1.88
Δ_f_*H*/kJ mol^−1^^[g]^	714.6	−55.5	−105.8	86.3	−188.9
*P/*GPa^[h]^	35.0	21.1	30.0	35.1	35.9
*v_*D*_/*m s^−1^^[i]^	8,875	7,017	7,982	8,823	9,000

[a] *Impact sensitivity*.

[b] *Friction sensitivity*.

[c] *Nitrogen and oxygen content*.

[d] *Oxygen balance assuming the formation of CO*.

[e] *Thermal decomposition temperature*.

[f] *Gas pycnometer (25°C)*.

[g] *Calculated enthalpy of formation*.

[h] *Detonation pressure*.

[i] *Detonation velocity*.

## Conclusions

A unique and facile approach to the nitro-free planar energetic compound 7-hydroxy-difurazano[3,4-b:3′,4′-f]furoxano[3″,4″-d]azepine (**4**) was presented. Interestingly, **4** and 7*H*-difurazano[3,4-*b*:3′,4′-*f*]furoxano[3″,4″-*d*]azepine (**3**) could be obtained only by controlling the reaction temperature, respectively. As expected, flat molecule structure with high nitrogen and oxygen content endow **4** high measured density (ρ = 1.92 g cm^−3^), high detonation properties (*v*_D_ = 8,875 m s^−1^, *P* = 35.0 GPa), and distinctly low sensitivities (IS: 21 J; FS > 360 N), which are good consistent with our design concept. The single-crystal X-ray structure of **4·**MeOH and **4·**H_2_O revealed the face-to-face stacking as well as edge-to-face arrangement, respectively. It is the face-to-face and edge-to-face assembly that plays a pivotal role in molecular density and sensitivity, and illustrate structure–property relationships. The intriguing properties of **4** provide an optional path to develop new high-performing insensitive materials.

## Data Availability

All datasets generated for this study are included in the manuscript/Supplementary Files.

## Author Contributions

BW conceived the experiment(s). LZ and HH conducted the experiment(s). YL and FB ran calculations. XL and SC revised and edited the manuscript.

### Conflict of Interest Statement

The authors declare that the research was conducted in the absence of any commercial or financial relationships that could be construed as a potential conflict of interest.
