# Supplementary material for: The Ingenious Synthesis of a Nitro-Free Insensitive High-Energy Material Featuring Face-to-Face and Edge-to-Face π-Interactions
Source: Front Chem. 2019 Aug 7;7:559. doi: 10.3389/fchem.2019.00559 (PMC6692488; doi:10.3389/fchem.2019.00559)
Supplement: Supplementary file 1 [file Data_Sheet_1.docx]

Supplementary Material

The Ingenious Synthesis of a Nitro-free Insensitive High-energy Material Featuring Face-to-face and Edge-to-face *π*-interactions

Lianjie Zhai^1^, Fuqiang Bi^1^, Huan Huo^1^, Yifen Luo^1^, Xiangzhi Li^1^, Sanping Chen^2^, Bozhou Wang ^1*^

^1^State Key Laboratory of Fluorine & Nitrogen Chemicals, Xi’an Modern Chemistry Research Institute, Xi’an, China

^2^College of Chemistry and Materials Science, Northwest University, Xi’an, China

*** Correspondence:**Bozhou Wang
wbz600@163.com

**Table of Contents**

**1 X-ray Crystallography**

**2 ^1^H and ^13^C NMR spectra**

**3 DSC curve**

**4 Geometry Coordinates**

**1 X-ray Crystallography**

**Table S1** Crystallographic data for **4·**MeOH and **4·**H_2_O.

|  | **4·**MeOH | **4·**H_2_O |
| --- | --- | --- |
| Empirical formula | C_7_H_5_N_7_O_6_ | C_6_H_3_N_7_O_6_ |
| Formula weight /g mol^−1^ | 283.18 | 269.15 |
| Temperature /K | 296(2) | 296(2) |
| Crystal system | Orthorhombic | Orthorhombic |
| Space group | *Pna*2_1_ | *Pnma* |
| Crystal size(mm) | 0.14 ×0.10× 0.10 | 0.15 × 0.14 × 0.13 |
| *a*(Å) | 10.5248(13) | 6.998(2) |
| *b*(Å) | 16.971(3) | 9.613(3) |
| *c*(Å) | 5.5806(9) | 14.788(5) |
| *α*(º) | 90 | 90 |
| *β*(º) | 90 | 90 |
| *γ*(º) | 90 | 90 |
| *V* (nm^3^) | 0.9968(3) | 0.9948(5) |
| Z | 4 | 4 |
| *D_c_* (g cm^−3^) | 1.887 | 1.797 |
| *μ* (mm^−1^) | 0.167 | 0.162 |
| F(000) | 576 | 544 |
| *θ* range for data collection | 3.084 to 28.302 | 2.53 to 26.35 |
| Dataset h | -14; 13 | -8; 8 |
| Dataset k | -22; 20 | -11; 11 |
| Dataset l | -5; 7 | -18; 16 |
| Reflections collected | 9838 | 5144 |
| Independent reflections | 1985 [*R*(int) = 0.0797] | 1079 [R(int) = 0.0283] |
| Completeness to 2*θ* | 99.8 % | 100.0 % |
| Data/restraints/parameters | 1785/ 1 / 184 | 1079 / 9 / 100 |
| Goodness-of-fit on *F*^2^ | 1.075 | 1.061 |
| *R*_1_, *wR*_2_ [*I*>2*σ*(*I*)] | 0.0664, 0.1550 | 0.0673, 0.0851 |
| *R*_1_, *wR*_2_ (all data) | 0.1165, 0.1767 | 0.1414, 0.1026 |
| Largest diff. peak and hole /e Å^−3^ | 0.429 and -0.298 | 0.175 and -0.223 |
| Solution | SHELXS-97 | SHELXS-97 |
| Refinement | SHELXL-97 | SHELXL-97 |
| Absorption correction | multi-scan | multi-scan |
| CCDC | 1911348 | 1911347 |

**2 ^1^H and ^13^C NMR spectra**

**
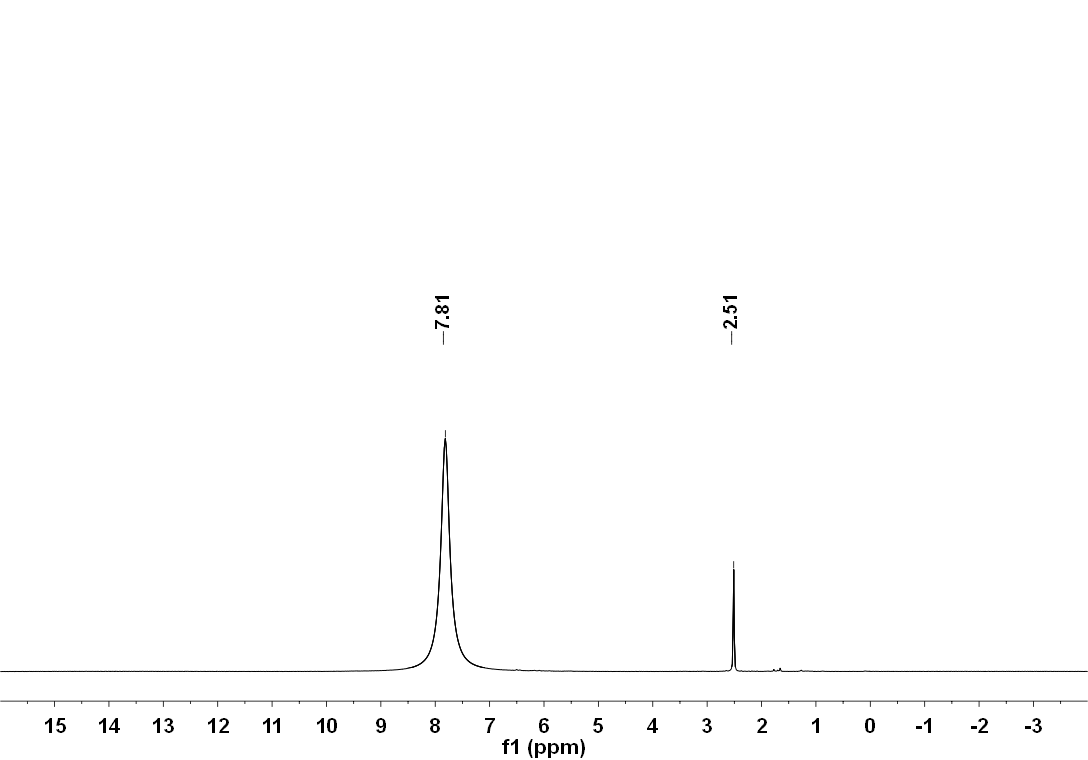
**

**Figure S1** ^1^H NMR spectrum of **4** in [D_6_]DMSO

**
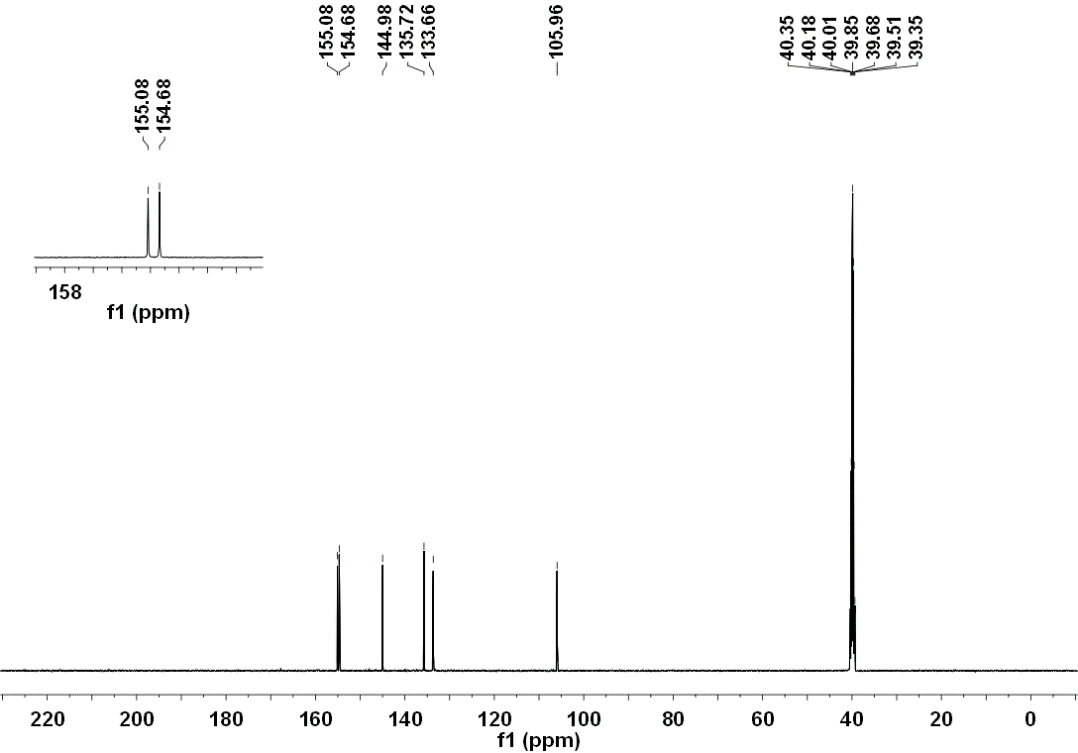
**

**Figure S2**  ^13^C NMR spectrum of **4** in [D_6_]DMSO

**3 DSC curve**


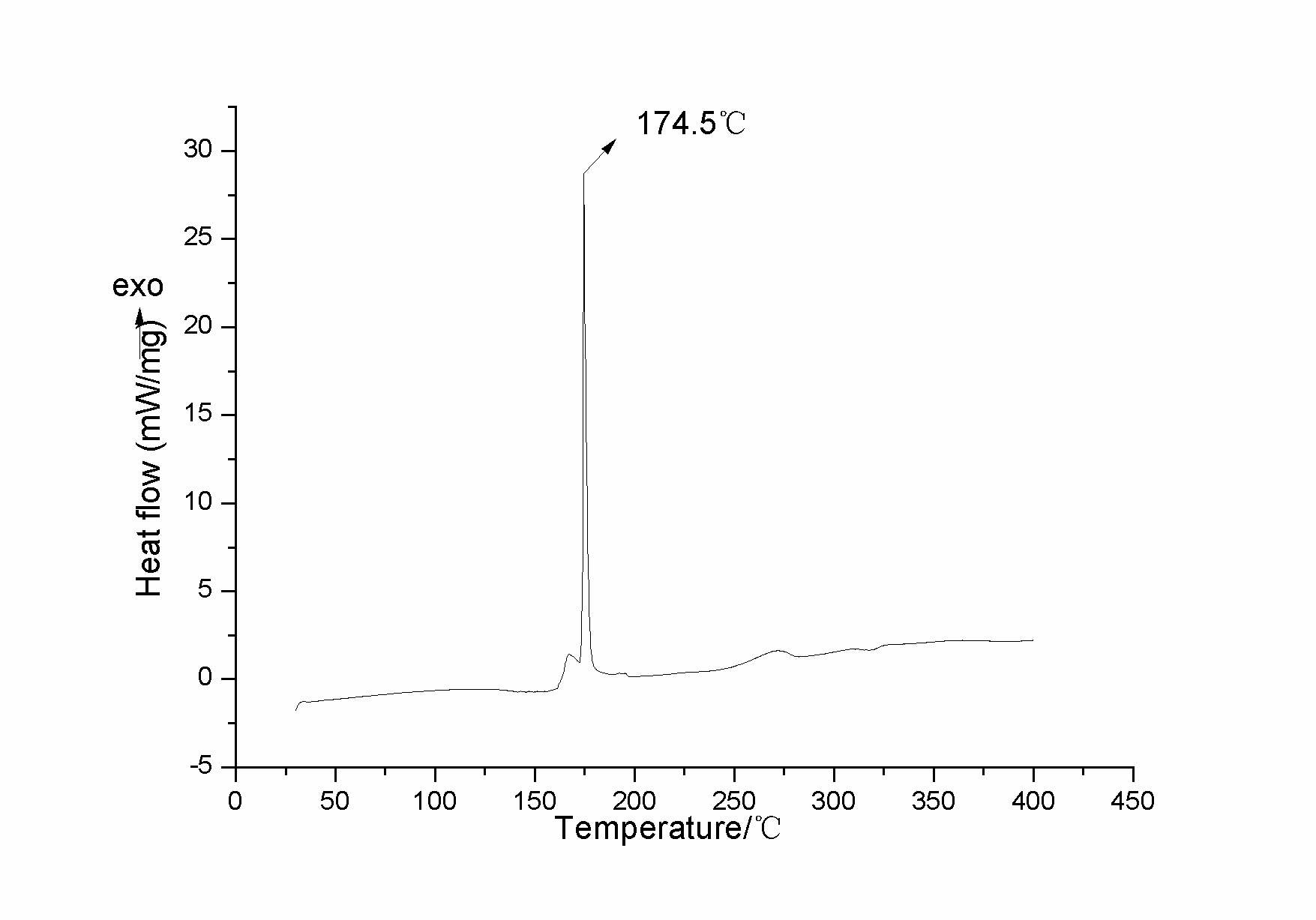


Figure S3 DSC curve of 4. The DSC plots were recorded at the heating rate of 5 °C·min^-1^.

**4 Geometry Coordinates**

**Cartesian Coordinates of Optimized geometries**


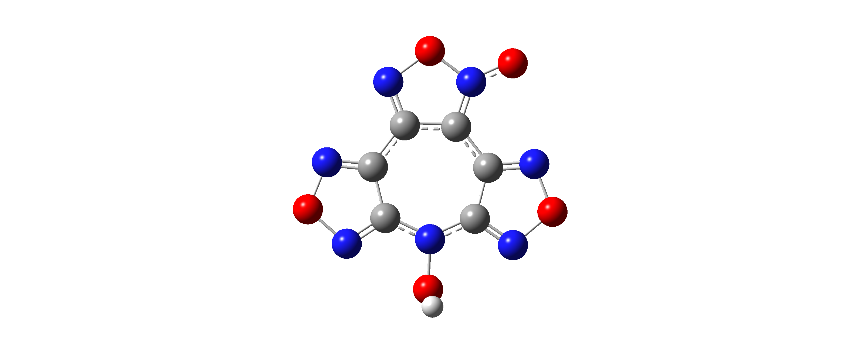


CBS-4M

CBS-4 Energy = -986.992421 Hartree＞

CBS-4 Enthalpy= -986.991477 Hartree

CBS-4 Free Energy=-987.044343 Hartree

| C | -0.26597800 | -1.49591600 | -0.00203200 |
| --- | --- | --- | --- |
| C | -1.26613900 | -0.47906600 | -0.00040900 |
| C | -1.12025200 | 0.94334500 | 0.01686400 |
| C | 0.11985900 | 1.70156600 | 0.02970100 |
| C | 1.89404500 | -0.03284000 | 0.02804200 |
| C | 1.16443400 | -1.28550600 | 0.01504000 |
| N | 3.15461300 | -0.26526900 | 0.04100500 |
| O | 3.27307600 | -1.69227500 | 0.03051900 |
| N | 1.99429800 | -2.25879800 | 0.01674400 |
| N | -0.77135400 | -2.67515000 | -0.02003000 |
| O | -2.19243700 | -2.48757600 | -0.03187300 |
| N | -2.42359700 | -1.08130100 | -0.01798300 |
| N | -2.11191000 | 1.75591300 | 0.02074500 |
| O | -1.56025700 | 3.05171500 | 0.03672400 |
| N | -0.13572500 | 2.95405500 | 0.04618500 |
| O | -3.60720800 | -0.65391800 | -0.02349400 |
| N | 1.40922500 | 1.24277300 | 0.05644100 |
| O | 2.37964800 | 2.23146200 | -0.27453300 |
| H | 2.69275500 | 2.58966800 | 0.57628200 |
